# Prevalence and correlates of the misuse of z-drugs and benzodiazepines in the National Survey on Drug Use and Health

**DOI:** 10.3389/fpsyt.2023.1129447

**Published:** 2023-03-09

**Authors:** R. Kathryn McHugh, Victoria R. Votaw, Emma W. Trapani, Megan D. McCarthy

**Affiliations:** ^1^Division of Alcohol, Drugs, and Addiction, McLean Hospital, Belmont, MA, United States; ^2^Department of Psychiatry, Harvard Medical School, Boston, MA, United States; ^3^Department of Psychology, University of New Mexico, Albuquerque, NM, United States; ^4^Center on Alcohol, Substance Use, and Addictions, University of New Mexico, Albuquerque, NM, United States

**Keywords:** benzodiazepines, z-drugs, sedatives, prescription drug misuse, sedative/anxiolytic use disorder

## Abstract

**Background:**

Benzodiazepines and non-benzodiazepine hypnotics (z-drugs) are commonly prescribed for their anxiolytic and hypnotic properties, though they can also be misused. In studies examining the epidemiology of prescription drug misuse, these medication classes are commonly combined, rendering inadequate knowledge of their patterns of misuse. The objective of this study was to characterize the population prevalence, conditional dependence, and sociodemographic and clinical correlates of the misuse of benzodiazepines and z-drugs.

**Methods:**

Data from the National Survey on Drug Use and Health from 2015 to 2019 were used to estimate population-level prevalence and characteristics of benzodiazepine and z-drug misuse. Groups were derived based on past-year misuse of benzodiazepines alone, z-drugs alone, or both drug types. Unadjusted regression analyses were used to compare groups on characteristics of interest.

**Results:**

Exposure to benzodiazepines and/or z-drugs *via* prescription or misuse was common; however, only 2% of the population was estimated to have misused a benzodiazepine in the past year, and less than 0.5% misused z-drugs. People who misused only z-drugs were generally older, more likely to have health insurance, more educated, and had less severe psychiatric symptoms. This group was also more likely to report misuse to cope with sleep difficulty. Although concurrent substance use was highly prevalent in all groups, people who misused z-drugs alone generally reported less concurrent substance use than the other groups.

**Conclusion:**

The misuse of z-drugs is less common than benzodiazepines, and people who misuse only z-drugs appear to generally have lower clinical severity. Nonetheless, a substantial subgroup of people exposed to z-drugs report concurrent, past-year use of other substances. Further research on z-drug misuse, including consideration of whether it should be grouped with other anxiolytic/hypnotic drugs, is needed.

## 1. Introduction

Benzodiazepines and non-benzodiazepine hypnotics (also referred to as z-drugs) are commonly prescribed for their anxiolytic and/or hypnotic effects. In addition to their therapeutic potential, these medications also have reinforcing properties (1–3) and thus can be misused (i.e., used at a dose or frequency greater than prescribed, without a prescription, or for reasons other than their therapeutic effect). Although these drugs have only modest reinforcing properties (2, 4–6), their misuse is common (7). The prevalence of misuse may be attributable—at least in part—to the high levels of population exposure to these drugs *via* prescription (8). Misuse of benzodiazepines and other sedatives can lead to an array of adverse consequences, such as the development of sedative/anxiolytic use disorder (9), and these medications are often present in drug overdose deaths, such as opioid overdoses (10).

Despite these public health impacts, little is known about differences in the misuse of benzodiazepines and z-drugs. The National Survey on Drug Use and Health (NSDUH), conducted annually by the Substance Abuse and Mental Health Services Administration (SAMHSA), is the largest epidemiological survey on substance use trends in the United States (U.S.). Within the NSDUH, sedatives (e.g., z-drugs and benzodiazepines with hypnotic effects) and tranquilizers (e.g., benzodiazepines with anxiolytic effects and non-benzodiazepine tranquilizers) are assessed separately, but these drugs are commonly combined into a single category of tranquilizing/sedating drugs in studies examining the epidemiology of prescription drug misuse (11–13). Similarly, even in studies outside of the NSDUH, investigators commonly assess the use and misuse of drugs producing anxiolytic and/or hypnotic effects as one category (14). Given differences in the mechanisms of action, therapeutic effect, and access to these medications, understanding differences in the populations at risk for misusing these medications as well as patterns of and reasons for misuse may help to support risk stratification and ultimately can begin to inform interventions for reducing misuse of these medications.

The overarching objective of this study was to characterize and compare the prevalence of z-drug and benzodiazepine misuse, as well as clinical correlates, past-year concurrent substance use, and motives for misusing medications utilizing annual population survey data from the NSDUH. Our first aim was to characterize the past-year prevalence of z-drug and benzodiazepine use and misuse. We also aimed to characterize the conditional misuse and dependence rates of these medications, which we defined as the proportion of people with *any* use of z-drugs or benzodiazepines in the past year (including use as prescribed or misuse) who misused these drugs or reported symptoms of a sedative/anxiolytic use disorder, respectively. Our second aim was to compare the sociodemographic and clinical characteristics of people who misused z-drugs and/or benzodiazepines in the last year. Our third aim was to characterize the past-year prevalence of other drug use and the extent of concurrent substance use, which we defined as the use of multiple substances over a defined period (15), among people who misused z-drugs and/or benzodiazepines in the last year. Finally, we aimed to compare motives for misuse among people who misused z-drugs and/or benzodiazepines. This was an exploratory, hypothesis-generating study.

## 2. Materials and methods

This secondary data analysis was preregistered on the Center for Open Science Framework.^1^ We analyzed data collected as part of the NSDUH between the years 2015 and 2019. The NSDUH is an annual population survey in the United States that assesses substance use and related health variables. The NSDUH assesses benzodiazepine and z-drug use and misuse separately and can be used to estimate population prevalence and associated characteristics.

The NSDUH is an independent, multistage probability sample for each of the 50 states and Washington, DC. Each year, approximately 70,000 individuals are asked to complete a screening survey. To be eligible to participate, individuals must be above 12 years of age and reside in the United States. Selected participants then move to an interview phase in which data are collected. Participants are compensated $30 for completing the interview. By aggregating data from 2015 to 2019, our sample includes 282,768 participants.

### 2.1. Measures

All variables were assessed using a standardized assessment administered by the Substance Abuse and Mental Health Services Administration. Details about the assessment are available at: https://www.samhsa.gov/data/data-we-collect/nsduh-national-survey-drug-use-and-health.

The NSDUH assesses the prevalence of use and misuse of four categories of prescription drugs, including opioid pain relievers, psychostimulants, tranquilizers, and sedatives. Although benzodiazepines are included in both the tranquilizer and sedative categories, they are also combined into a separate category of “any benzodiazepine,” which was used for these analyses. The z-drugs, including zolpidem, eszopiclone, and zaleplon (both generic and brand name), are exclusively assessed within the sedative category. To derive variables for z-drug use and misuse, we combined each of the assessed z-drugs into one category.

Binary (yes/no) indicators of any past-year use (including use as prescribed and misuse) and misuse only were used to define three groups: benzodiazepines only, z-drugs only, and combined benzodiazepines and z-drugs. The NSDUH collects data on any use, including use as prescribed and misuse, for certain benzodiazepines, including alprazolam products, lorazepam products, clonazepam products, diazepam products, temazepam products, flurazepam, or triazolam; if a participant reports past-year misuse of any additional benzodiazepine, they were also coded as reporting any past-year use. These data are collected by displaying the names and pictures of various benzodiazepines and asking respondents to indicate which medications they have used in the past year in any form. NSDUH also collects data on misuse, which is defined as the use of prescription drugs “in any way that a doctor did not direct you to use them…including taking someone else’s prescription, or taking one’s own prescription in any way other than prescribed (e.g., taking a larger quantity, taking more often, or taking for a longer duration than prescribed)”(16).

To assess the prevalence of sedative/anxiolytic use disorder (the substance use disorder corresponding to the problem use of benzodiazepines or z-drugs), we used a binary (yes/no) indicator of the presence of a past-year *Diagnostic and Statistical Manual of Mental Disorders, 4th Edition (DSM-IV)* (17) diagnosis of abuse or dependence for sedatives or tranquilizers (i.e., anxiolytics). Of note, the NSDUH assessed *DSM-IV* abuse and dependence symptoms separately. We combined these in the present analysis, roughly consistent with the *DSM-5* (18) diagnosis of sedative, hypnotic, or anxiolytic use disorder.

In addition to demographic data provided by the NSDUH, indicators of mental health, overall health, and functioning were also included to characterize differences in drug type groups. To assess suicidal thinking and behavior, all participants over 18 years of age were asked if, in the past year, they had serious thoughts of killing themselves (suicidal thinking), made a plan to kill themselves (suicide plan), or attempted to kill themselves (suicide attempt) (19). Participants were asked to provide a binary (yes/no) response. The Kessler-6 Distress Scale was used to measure psychological distress. Participants received a score from 0 to 24, with higher scores reflecting greater psychiatric distress (20). The World Health Organization Disability Assessment Schedule (WHODAS) was used to assess impaired functioning in various domains such as cognition, mobility, and self-care (21). The possible total scores range from 0 to 24, with higher scores reflecting more severe impairment. A single item assessing self-reported overall health, ranging from “poor” to “excellent,” was used as an indicator of health status.

### 2.2. Analysis

Unadjusted population prevalence was estimated for the misuse of z-drugs and/or benzodiazepines first in the full sample and in the subgroup of people who reported *any* use (prescribed or misused) of these drugs in the past year. The unadjusted prevalence of sedative/anxiolytic use disorder was also estimated in both the full sample and the subgroup who reported past-year use of z-drugs and/or benzodiazepines. In addition, multinomial logistic regression analyses were used to compare the likelihood of reporting misuse or a use disorder across three groups of respondents with *any* z-drug or benzodiazepine use (z-drug use only, benzodiazepine use only, use of both z-drugs and benzodiazepines).

Multinomial logistic regressions were used to compare sociodemographic and clinical characteristics among the three groups (z-drug misuse only, benzodiazepine misuse only, and misuse of both z-drugs and benzodiazepines), with group status as the dependent variable. These characteristics included: gender, health insurance status, age, race, education, suicidal thinking and behavior (suicidal thinking, suicide plan, suicide attempt), overall health, functional impairment, and psychiatric distress.

Unadjusted multinomial logistic regression analyses also were used to compare misuse groups with respect to the presence of past-year use of other substances, including alcohol, tobacco, heroin, cocaine (combined crack and powder cocaine), methamphetamine, hallucinogens, inhalants, misused opioid analgesics, and misused stimulants, as well as the count of the total number of substances used.

Finally, unadjusted multinomial logistic regression was used to compare misuse groups with respect to each motive for the last episode of tranquilizer and/or sedative misuse. As multiple motives could be reported (i.e., categories were not mutually exclusive), separate regressions were conducted for each motive.

Consistent with prior investigations using this dataset, we used an alpha of 0.05 for significance testing. A false discovery rate procedure (22) adjusted the *p*-value for multiple testing for each outcome. All analyses accounted for the complex survey design of the study (i.e., oversampling of young adults and racial/ethnic minority populations) and the use of combined years of survey data following the recommendations from SAMHSA.

## 3. Results

*Any* use of benzodiazepines or z-drugs (including use as prescribed and misuse) was highly prevalent, with an estimated 13.7% of the population using one of these medications in the previous year. Benzodiazepine use was more than twice as common as z-drug use, with an estimated 11.4% of the population using or misusing a benzodiazepine and 4% using or misusing a z-drug.

### 3.1. Misuse prevalence estimates

**FIGURE 1:**
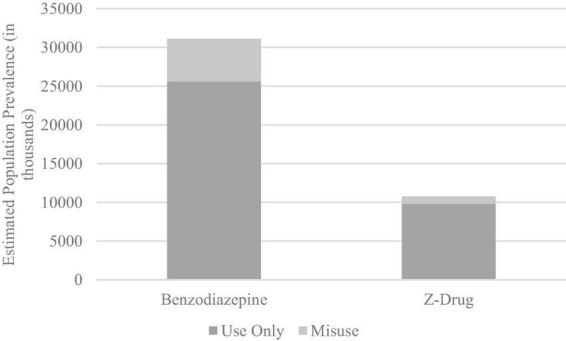
Estimated prevalence of prescribed use only and misuse of benzodiazepines and z-drugs (in thousands).

The past-year prevalence of benzodiazepine and z-drug misuse is depicted in Figure 1. An estimated 2% of the population engaged in past-year misuse of a benzodiazepine, and less than 0.4% misused a z-drug. The prevalence was much higher among people exposed to these medications in the past year (including legitimate use as prescribed), with an estimated 17.7% of all people who used benzodiazepines misusing them and 9.2% of people who used z-drugs misusing them.

The proportion of people with a sedative/anxiolytic use disorder was similar between drug types, with 2.3% of people who used benzodiazepines and 2.1% of people who used z-drugs reporting a past-year sedative/anxiolytic use disorder. Among people who reported past-year misuse of benzodiazepines, 12.3% met criteria for a sedative/anxiolytic use disorder, and 13.6% of people who reported past-year z-drug misuse met criteria for a disorder.

When considering subgroups of participants based on whether, in the past year, they exclusively used benzodiazepines, exclusively used z-drugs, or used both, the prevalence of misuse and use disorder varied. Any past-year use of *both* z-drugs and benzodiazepines was associated with the highest likelihood of misuse (compared to benzodiazepines alone: OR = 1.36, 95% CI = 1.23, 1.51; compared to z-drugs alone OR = 3.64, 95% CI = 3.09, 4.29), followed by benzodiazepines alone (compared to z-drugs: OR = 2.67, 95% CI = 2.23, 3.07), and finally z-drugs alone.

This same pattern of findings was observed for sedative/anxiolytic use disorder. Specifically, people who used both drug types were more likely to meet criteria for a sedative/anxiolytic use disorder than those who used benzodiazepines alone (OR = 2.42, 95% CI = 1.91, 3.07) or z-drugs alone (OR = 13.89, 95% CI = 7.78, 24.79); benzodiazepine use alone was also associated with higher odds of a use disorder than z-drugs alone (OR = 5.74, 95% CI = 3.18, 10.36).

### 3.2. Sociodemographic and clinical characteristics

The sociodemographic and clinical characteristics of the three groups (past-year z-drug misuse only, benzodiazepine misuse only, both z-drug and benzodiazepine misuse) are presented in Table 1. These analyses indicated significant overall differences among groups in age, education, and health insurance status, but not gender. In general, these results demonstrated a pattern of older age for people with z-drug use only (approximately 40% of people who reported only misusing z-drugs were 50 or older). The z-drug-only group also generally had higher levels of education, partly due to the low base rate in school-aged adolescents, which was less than half of the adolescent prevalence rate of the benzodiazepine-only and combined groups. Finally, the z-drug-only group had very high rates of health insurance, with over 94% reporting health insurance, compared to 85% of benzodiazepine use only and 86% in the combined group.

**TABLE 1:** Population estimates of sociodemographic characteristics of people with past-year benzodiazepine and/or z-drug misuse.

	Benzodiazepine misuse only		Z-drug misuse only		Benzodiazepine and z-drug misuse	
	**Estimate**	**(95% CI lower, upper)**	**Estimate**	**95% CI**	**Estimate**	**95% CI**
**Age^a^**						
12–17 years old	7.5%	(6.9, 8.1%)	3.5%	(2.5, 4.9%)	7.4%	(5.0, 11.0%)
18–25 years old	30.3%	(28.8, 31.7%)	12.8%	(10.4, 15.5%)	23.5%	(19.5, 28.0%)
26–34 years old	23.3%	(21.9, 24.7%)	19.9%	(16.3, 24.2%)	22.1%	(17.2, 28.0%)
35–49 years old	18.8%	(17.6, 20.0%)	24.0%	(18.7, 30.4%)	21.0%	(15.6, 27.6%)
50–64 years old	15.0%	(13.2, 17.0%)	28.5%	(22.6, 35.3%)	21.2%	(14.4, 30.1%)
65 or older	5.2%	(4.1, 6.7%)	11.3%	(7.3, 17.1%)	4.8%	(1.9%, 11.5%)
**Gender**						
Male	50.3%	(48.6, 51.9%)	43.5%	(37.9, 49.3%)	49.7%	(44.0, 55.4%)
Female	49.7%	(48.1, 51.4%)	56.5%	(50.7, 62.1%)	50.3%	(44.6, 56.0%)
**Race^b^**						
White (non-Hispanic)	73.3%	(71.2, 75.3%)	75.6%	(70.2, 80.2%)	82.9%	(78.0, 86.9%)
Black/African American (non-Hispanic)	7.2%	(6.5, 8.0%)	5.9%	(3.5, 10.0%)	3.2%	(1.6, 6.2%)
Native American/Alaska native (non-Hispanic)	0.3%	(0.3, 0.5%)	0.5%	(0.2, 1.6%)	0.6%	(0.2, 2.3%)
Native Hawaiian/other Pacific Islander (non-Hispanic)	0.3%	(0.2, 0.5%)	*	*	0.1%	(0.0, 1.1%)
Asian (non-Hispanic)	1.8%	(1.3, 2.5%)	3.1%	(1.5, 6.2%)	1.4%	(0.7, 2.8%)
More than 1 race (Non-Hispanic)	2.5%	(2.1, 3.0%)	3.0%	(1.7, 5.2%)	2.1%	(1.3, 3.5%)
Hispanic	14.5%	(12.9, 16.3%)	11.9%	(7.9, 17.7%)	9.7%	(6.4, 14.3%)
**Education^a^**						
Less than high school	10.7%	(9.5, 12.0%)	5.1%	(3.4, 7.5%)	8.8%	(5.5, 13.6%)
High School grad	21.5%	(20.0, 23.0%)	15.1%	(11.5, 19.6%)	22.4%	(17.8, 27.7%)
Some college/associates degree	36.7%	(35.0, 38.4%)	35.4%	(29.5, 41.8%)	26.8%	(21.5, 32.7%)
College graduate	23.6%	(22.2, 25.1%)	40.9%	(35.4, 46.6%)	34.7%	(28.6, 41.2%)
12–17 years olds	7.5%	(6.9, 8.1%)	3.5%	(2.5, 4.9%)	7.4%	(5.0, 11.0%)
**Health insurance^a^**						
Yes, respondent is covered by health insurance	84.8%	(83.5, 86.0%)	94.1%	(91.0, 96.2%)	86.1%	(80.4, 90.3%)
No, respondent is not covered by health insurance	15.2%	(14.0, 16.5%)	5.9%	(3.8, 9.0%)	13.9%	(9.7, 19.6%)
**Serious suicidal ideation^a^**						
No	81.7%	(80.2, 83.1%)	89.4%	(85.6, 92.3%)	75.1%	(69.3, 80.1%)
Yes	18.3%	(16.9, 19.8%)	10.6%	(7.7, 14.4%)	24.9%	(19.9, 30.7%)
**Suicide plan^a^**						
No	92.7%	(91.7, 93.6%)	96.6%	(94.0, 98.1%)	89.0%	(83.8, 92.7%)
Yes	7.3%	(6.4, 8.3%)	3.4%	(1.9, 6.0%)	11.0%	(7.3, 16.2%)
**Suicide attempt^a^**						
No	96.3%	(95.6, 96.9%)	99.1%	(98.5, 99.5%)	93.0%	(89.2, 95.6%)
Yes	3.7%	(3.1, 4.4%)	0.9%	(0.5, 1.5%)	7.0%	(4.4, 10.8%)
Kessler-6 score	10.67	(10.38, 10.95)	8.52	(7.80, 9.23%)	11.70	(10.41, 12.99)
WHODAS score	8.45	(8.14, 8.76)	7.40	(6.62, 8.19%)	9.93	(8.72, 11.14)
**Overall health**						
Excellent	15.7%	(14.2, 17.2%)	21.0%	(16.6, 26.3%)	21.0%	(15.5, 27.6%)
Very good	36.7%	(34.9, 38.5%)	40.8%	(34.8, 47.1%)	33.9%	(27.1, 41.5%)
Good	32.3%	(30.5, 34.1%)	25.5%	(20.1, 31.9%)	24.9%	(19.1, 31.8%)
Fair/poor	15.4%	(14.0, 16.9%)	12.7%	(8.9, 17.7%)	20.2%	(14.9, 26.8%)

^a^Overall model was statistically significant using false discovery rate correction (*p* ≤ 0.009). ^b^Significance not reported due to small cell sizes, *data not available. Although a small number of participants in the z-drug-only group reported past-year use of non-benzodiazepine tranquilizers, data on the source for those drugs are not presented. WHODAS, World Health Organization Disability Assessment Schedule.

Descriptive data are presented for race in Table 1; however, the regression results were not interpreted due to low base rates for some combinations of race and substance use resulting in quasi-complete separation in the regression models.

With respect to clinical characteristics, model effects were found for suicidal ideation, suicide plan, and suicide attempt, as well as psychiatric distress and disability, but not for overall health status. Suicidal thinking and behavior were consistently most common in the combined group (24.9% estimated to have suicidal ideation, 11% suicide plan, 7% suicide attempt), followed by benzodiazepines only (18.3% suicidal ideation, 7.3% suicide plan, 3.7% suicide attempt) and z-drugs only (10.6% suicidal ideation, 3.4% suicide plan, 0.9% suicide attempt). This is consistent with results of the psychiatric distress (Kessler-6) and disability (WHODAS) results, which found that scores were highest in the combined group, followed by the benzodiazepine-only group, and finally, the z-drug-only group.

### 3.3. Other drug use and concurrent substance use

Other drug use was highly prevalent in all three groups. Drug use also varied between groups, with some variability in the magnitude of effects (Table 2). The benzodiazepine and z-drug group consistently reported more drug use than the z-drug alone group for all substances except alcohol, which was common (>85%) in all three groups. The combined group also was more likely to report past-year use of all drugs than the benzodiazepine-only group except alcohol, tobacco, and cannabis. The benzodiazepine-only group reported more past-year substance use for all substances than the z-drug-only group, except for alcohol and inhalants.

**TABLE 2:** Estimated population prevalence of past-year substance use among people reporting past-year benzodiazepine and z-drug misuse.

	Benzodiazepine misuse only		Z-drug misuse only		Benzodiazepine and z-drug misuse	
	**Estimate**	**(95% CI lower, upper)**	**Estimate**	**95% CI**	**Estimate**	**95% CI**
Alcohol	88.7%	(87.0, 90.2%)	85.3%	(80.6, 88.9%)	87.2%	(81, 91.5%)
Tobacco^a^	65.6%	(63.7, 67.4%)	40.5%	(34.8, 46.5%)	73.8%	(63.3, 82.1%)
Marijuana^a^	63.7%	(61.8, 65.5%)	37.0%	(32.1, 42.2%)	67.9%	(59.9, 74.9%)
Heroin^a^	5.4%	(4.6, 6.2%)	1.1%	(0.5, 2.2%)	12.0%	(8.7, 16.4%)
Cocaine or crack^a^	3.7%	(3.0, 4.4%)	1.0%	(0.3, 3.5%)	8.2%	(5.2, 12.8%)
Hallucinogens^a^	21.6%	(20.4, 22.9%)	7.5%	(6.1, 9.3%)	29.5%	(24.6, 35.0%)
Inhalants^a^	5.4%	(4.6, 6.3%)	4.1%	(2.0, 8.1%)	9.7%	(7.3, 12.9%)
Methamphetamine^a^	8.2%	(7.4, 9.2%)	1.1%	(0.4, 2.9%)	14.1%	(10.8, 18.3%)
Pain relievers^a^	42.2%	(40.3, 44.0%)	28.5	(23.2, 34.5%)	65.2%	(58.8, 71.1%)
Stimulants^a^	24.9%	(23.6, 26.3%)	10.9%	(8.1, 14.6%)	38.2%	(31.7, 45.0%)

^a^Overall model was statistically significant using false discovery rate correction (*p* ≤ 0.002).

Consistent with these findings, more concurrent substance use (count of substances used, including sedatives and tranquilizers) was greater in the combined group (estimated population mean = 6.03 drugs, 95% CI = 5.70, 6.37) than in the z-drug-only group (mean = 3.19, 95% CI = 3.05, 3.33) and the benzodiazepine-only group (mean = 4.32, 95% CI = 4.25, 4.39), and greater in the benzodiazepine-only group than in the z-drug only group.

### 3.4. Motives for misuse

**FIGURE 2:**
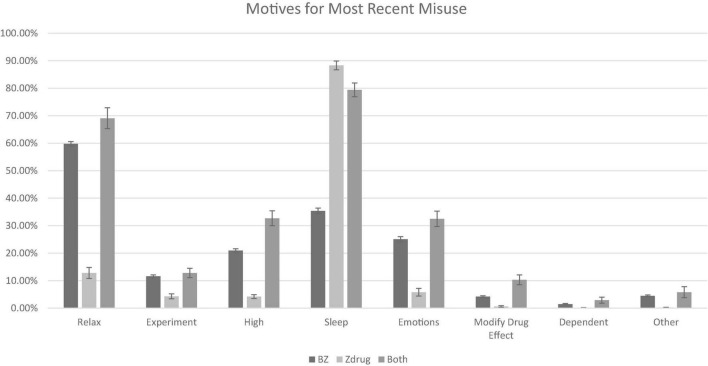
Motives for most recent misuse (*modify drug effect* refers to attempts to increase or decrease the effects of another substance; *dependent* refers to physiological dependence). All model effects statistically significant using false discovery rate correction (*p* ≤ 0.002). BZ, benzodiazepine only group.

Motives for the most recent misuse also varied significantly across groups (see Figure 2). People who misused z-drugs only were less likely than both the combined group and the benzodiazepine-only group to report all motives except for sleep. Misusing for sleep was significantly more common in the z-drug group than in the benzodiazepine group only (OR = 13.80, 95% CI = 9.98, 19.08) and was not significantly different from the combined group (OR = 1.96, 95% CI = 1.23, 3.13).

## 4. Discussion

The findings of the present analysis indicate that more than 1 in 10 people in the U.S. are exposed to either benzodiazepines or z-drugs annually. Although most of those exposed to these medications do not misuse them, a substantial subgroup reported misuse (approximately 9% of those exposed to z-drugs and almost 18% of those exposed to benzodiazepines), and a small subgroup reported misuse at the severity of a substance use disorder (approximately 2% of people exposed to either drug). Of note, the sedative/anxiolytic use disorder rates among those with any benzodiazepine use reported herein were similar to those previously estimated using 2015–2016 NSDUH data (9), but, to our knowledge, use disorder estimates have not been previously reported for those with z-drug use.

Our estimate for the prevalence of benzodiazepine misuse is similar to prior reports in the literature (9), indicating that approximately 2% of the U.S. population engages in misuse each year. Z-drug misuse has been less well-characterized, and notably, its prevalence was low in the general population (<0.05%). Nonetheless, more than 9% of people who reported any past-year use of a z-drug reported misuse, suggesting that misuse among people exposed to z-drugs is not uncommon.

Importantly, several differences were observed in the characteristics of people who misused these two drugs. When comparing people who misused benzodiazepines only and those who misused z-drugs only, people who misused z-drugs were generally older, more highly educated, and had substantially less severe psychiatric symptoms. The z-drug-only group was also less likely to report the use of most other drugs and had less concurrent substance use (i.e., fewer drug types used in the past year) than the benzodiazepine-only group. Previous research has also demonstrated that benzodiazepines have greater misuse liability among those with histories of alcohol use disorder (23) and increase the reinforcing effects of opioids when they are taken in combination (24), consistent with our findings that those with benzodiazepine misuse had higher rates of other drug use.

Yet, it is of note that the use of other drugs was common—and higher than general population base rates—in all three groups, including people who misused z-drugs alone. This pattern of concurrent substance use is concerning, given the potential for adverse events when these drugs are combined with other depressants. We are limited in our conclusions regarding the level of risk associated with concurrent substance use identified in our analysis, given data are not available on co-use or simultaneous use (e.g., using benzodiazepines and opioids at the same time), which is particularly risky for overdose. It has been thoroughly documented that simultaneous use of other substances, particularly opioids, is common among those who misuse benzodiazepines (14). However, future research on co-use among those who misuse z-drugs will be needed to better understand the prevalence of risky co-use patterns in this population.

Another future direction concerning polysubstance use might include characterizing co-occurring substance use disorders among those with z-drug misuse. We chose to focus on concurrent substance use broadly, given the risks associated with combining central nervous system depressants and the aim of our manuscript to inform risk stratification and methodological decisions regarding the measurement of benzodiazepine and z-drug misuse. However, there is evidence that other substance use disorders are highly prevalent among those with benzodiazepine misuse (9), and that benzodiazepine misuse is associated with poorer substance use disorder treatment outcomes (14). It is currently unclear the extent to which z-drug misuse co-occurs with other substance use disorders and impacts treatment outcomes.

The most common motive for the misuse of z-drugs—by far—was to sleep (over 88% of participants reported this motive). In contrast, misuse of benzodiazepines, or both benzodiazepines and z-drugs, was associated with a broad array of motives, such as to relax, to sleep, to manage emotions, to get high, to experiment, or to modify the effects of other drugs (e.g., to increase or decrease an effect of another substance). These differences are consistent with the mechanism of action and pharmacological properties of benzodiazepines and z-drugs. Z-drugs bind preferentially to GABA_*A*_ receptors containing α1-subunits, which modulate sedation and amnesia (25, 26). In contrast, benzodiazepines bind non-selectively to sites that contain α 1-, α 2-, α 3-, or α5-subunits, with the α2- and α3-subunits implicated in anxiolytic effects (25, 26).

Nevertheless, it is notable that the most common motives for both drug classes were consistent with *relief* motives rather than *reward* motives for misuse. A large body of literature indicates that negative reinforcement motives are associated with the progression of substance use severity (27, 28), and therefore assessing reasons for misuse of benzodiazepines and z-drugs might help identify individuals at risk of developing symptoms of a use disorder and target interventions addressing underlying problems with sleep and anxiety. Psychometric and qualitative research on motives for benzodiazepine and z-drug misuse are needed to further this line of work, given limited work in this area (14) and a lack of wide-spread measures of motives for benzodiazepines and z-drugs, such as is available for alcohol use motives [e.g., Drinking Motives Questionnaire (29)]. The assessment of motives for benzodiazepines and z-drug misuse likely requires unique considerations; for example, it is unclear the extent to which specific motives for misuse of these medications are distinct, given the overlap between items such as “to sleep” and “to relax.”

The results of the present analysis (e.g., differences in conditional misuse rates, motives) may be attributable to differences in the reinforcing properties of these two drug classes. Yet, few studies have directly compared the reinforcing properties of benzodiazepines and z-drugs, and extant studies were conducted over two decades ago, primarily enrolled men, and have produced equivocal findings. These human laboratory studies indicate that benzodiazepines (i.e., triazolam, alprazolam) and z-drugs (i.e., zolpidem, zopiclone) have similar reinforcing properties, as indicated by subject-rated measures (e.g., drug liking, street value) and drug choice paradigms (30–32). However, in these studies, z-drugs were more likely than benzodiazepines to produce adverse side effects (e.g., dizziness) and less likely to be identified as barbiturates, benzodiazepines, or alcohol in drug discrimination paradigms (30–32). Using a proposed algorithm to address the misuse liability of hypnotic drugs based on human laboratory findings in combination with other factors (e.g., half-life, actual misuse prevalence rates, severity of withdrawal), Griffiths and Johnson (5) concluded that the evaluated benzodiazepines generally had higher misuse liability than the evaluated z-drugs. Future research should directly compare the reinforcing properties of commonly misused benzodiazepines and z-drugs in more diverse samples.

Taken together, the results of the present analysis may help inform the decision on whether to combine anxiolytic and hypnotic drugs in future research and surveillance efforts. Our results suggest differences between z-drugs and benzodiazepines, ranging from conditional misuse and dependence rates to indicators that the groups that misuse z-drugs alone vs. benzodiazepines (with or without z-drugs) are less clinically severe, more likely to misuse for the drug’s indication (sleep), and report less concurrent substance use. Accordingly, studies that combine z-drugs and benzodiazepines may lead to underestimates of conditional liability and clinical severity of benzodiazepine misuse. This is not to say that z-drugs are free of potential harm, as they increase the risk of overdose in high-risk populations (33), but accurately characterizing the population of those most likely to misuse z-drugs might help inform preventative efforts to reduce such harm. Ultimately, the decision whether to combine these drug types will depend on the question or interest and may be informed by statistical power (particularly for studies of z-drug misuse), we recommend that studies combining these drug types also include sensitivity analyses examining whether results of the combined group hold for each subpopulation.

## 5. Limitations and future directions

Several methodological limitations impact the interpretation of the present findings. First, this analysis is subject to the general limitations of the NSDUH, including the inability to generalize to groups un- or underrepresented (e.g., incarcerated people, un-housed people) and sampling biases (34). Several methodological features of the NSDUH (e.g., not assessing certain variables, not assessing variables over a past-year time frame) limited the variables we could examine in the present analysis. Several unexamined factors in the present analysis, such as co-use of substances, frequency of misuse, and source of prescription medications for misuse, would undoubtedly aid in the clarification of differences between those who use and misuse benzodiazepines and/or z-drugs. We also combined responses across tranquilizers and sedatives for several substance-specific variables, such as motives and use disorder, thus rendering conclusions about the unique effect of medication classes challenging. This is particularly relevant for the group who reported misuse of benzodiazepines and z-drugs, for which we cannot determine if use disorder was secondary to one or both of these substances and if those in this subgroup reported different motives for the misuse of different medication classes. Similarly, non-benzodiazepine tranquilizers and sedatives could contribute to participants’ responses to questions about motives and sedative/anxiolytic use disorder if a participant reported misuse of both benzodiazepine and non-benzodiazepine tranquilizers and/or sedatives, but given the low base rates of non-benzodiazepine products (35), this is likely exceedingly rare. We also decided to combine *DSM-IV* sedative/anxiolytic abuse and dependence, consistent with a DSM-5 approach to diagnosis. Nevertheless, this approach may have conflated complex persistent benzodiazepine dependence with sedative/anxiolytic use disorder, despite different treatment needs for these presentations (36). We recommend that future analyses of NSDUH data leverage latent variable mixture models to understand subtypes of sedative/anxiolytic use disorder symptoms (37), as well as item response theory to assess the validity of this diagnosis (38). Lastly, although the NSDUH 2020 public use data file is currently available, we excluded this data from our analysis, given substantially different substance use disorder prevalence rates in 2020 compared to previous years, which is likely attributable to the introduction of DSM-5 criteria and/or COVID-19 impacts on data collection procedures (39).

## 6. Conclusion

Overall, this study indicated that misuse is not uncommon in people exposed to benzodiazepines and z-drugs and should be monitored in people prescribed these medications. People who misuse both drug types appear to have significant clinical severity concerning psychiatric severity and other drug use. Those with concurrent substance use may be of particular concern for overdose, given the risks of combining substances, as well as elevated suicidal thinking and behavior. The populations of people misusing and motives for misuse further suggest differences between the misuse of benzodiazepines and z-drugs that may benefit from consideration, where possible, as separate categories.

## Data availability statement

Publicly available datasets were analysed in this study. This data can be found here: SAMHSA (datafiles.samhsa.gov).

## Author contributions

RM, VV, and MM planned the study aims and analyses. RM conducted the data analysis. All authors contributed to the draft of the manuscript and edited and approved the final manuscript.

## Footnotes

1
https://osf.io/vm658
